# Determinants of Menstrual Hygiene Practices Among Adolescent Schoolgirls in Saudi Arabia: Implications for Adolescent Health Promotion

**DOI:** 10.3390/healthcare14020171

**Published:** 2026-01-09

**Authors:** Aziza Ibrahim Mohamed, Amani Mahmoud Fadul, Ohood Ali Alkaabi, Mohammed Hassan Moreljwab, Eltayeb Abdelazeem Idress, Thuria Edrees Alhassan, Eman Elsayed Hussien Mohammad, Shereen Ahmed Elwasefy, Rabab Gad Abd El-Kader, Basma Maher Ragheb, Ramya Shine Aneesh

**Affiliations:** 1 Department of Nursing, College of Applied Medical Sciences, University of Bisha, Bisha 67714, Saudi Arabia; amokhtar@ub.edu.sa (A.M.F.); aaalkabi@ub.edu.sa (O.A.A.); mhassan@ub.edu.sa (M.H.M.); ehamed@ub.edu.sa (E.A.I.); thomyssara@gmail.com (T.E.A.); ehussein@ub.edu.sa (E.E.H.M.); 2 Medical Surgical Nursing, College of Nursing, University of Khartoum, Khartoum 13314, Sudan; 3 Medical Surgical Nursing, College of Nursing, University of Gadarif, Gadarif 32210, Sudan; 4 Nursing Department, College of Applied Medical Sciences, Jouf University, Sakakah 72388, Saudi Arabia; selwasifi@ju.edu.sa; 5 Rak College of Nursing, RAK Medical and Health Science University, Ras Al Khaimah Emirate 11172, United Arab Emirates; rabab@rakmhsu.ac.ae (R.G.A.E.-K.); basma@rakmhsu.ac.ae (B.M.R.); ramyashine@rakmhsu.ac.ae (R.S.A.); 6 Faculty of Nursing, Mansoura University, Mansoura 35516, Egypt

**Keywords:** menstrual hygiene, adolescent girls, determinants, Saudi Arabia

## Abstract

**Background**: Menstrual hygiene management (MHM) is a vital aspect of adolescent girls’ health and well-being. However, in many Gulf countries, including Saudi Arabia, this issue has received less attention because of cultural taboos, misconceptions, and a lack of knowledge about factors affecting menstrual hygiene practices. Thus, it is crucial to promote adolescent health and develop effective school-based interventions. **Aim of the Study**: Our aim was to assess menstrual hygiene practices and their determinants among adolescent girls in secondary schools in Bisha, Saudi Arabia. **Methods**: A cross-sectional descriptive approach was used to study 320 female secondary school students in Bisha City, southwestern Saudi Arabia. **Sample**: The subjects were selected by using a stratified random sampling procedure. Information was obtained using a previously validated and culturally sensitive self-administered questionnaire on knowledge and menstrual hygiene. Descriptive statistics, chi-square tests, and binary logistic regression analyses were conducted to determine factors associated with good menstrual hygiene practices. **Results**: A total of 320 adolescent girls participated, of whom 53.8% demonstrated good menstrual hygiene practices. In the multivariable analysis, independent predictors of good practices were increased age (AOR = 2.69, 95% CI:1.59–4.56), urban residency (AOR = 2.62, 95% CI: 1.46–4.69), and good menstrual knowledge (AOR = 2.13, 95%CI: 1.24–3.67). Maternal primary education (AOR = 8.033, CI: 1.44–44.99) and maternal employment in the government sector (AOR = 7.346, CI: 2.29–23.54) also showed strong positive associations with good menstrual hygiene practices. Conversely, experiencing menarche after age 12 was associated with lower odds of good menstrual hygiene (AOR = 0.49). **Conclusions**: Although a good proportion of girls practiced adequate menstrual hygiene, major knowledge and behavior gaps persist. Providing strong menstrual education in schools and through community-based efforts is critical for supporting the health of adolescent girls and promoting menstrual equity in Saudi Arabia.

## 1. Introduction

Menstrual hygiene management (MHM) is recognized as a significant global public health issue, as inadequate menstrual hygiene practices can lead to infections, school absenteeism, and adverse psychosocial outcomes among adolescent girls [1,2]. Despite global efforts to improve menstrual health through school-based interventions and public awareness programs, many adolescent girls—particularly in low- and middle-income countries—continue to face barriers such as limited access to sanitary products, insufficient knowledge, and persistent cultural stigma surrounding menstruation [3,4].

Menstrual hygiene management (MHM) in this study is defined according to the UNICEF Guidance on Menstrual Health and Hygiene and FIGO recommendations, encompassing the use of clean materials, appropriate disposal, regular personal hygiene, and adequate knowledge and attitudes that allow girls to manage menstruation safely and with dignity [1,2]. Menstrual health extends beyond the biological process of menstruation and includes physical, mental, and social well-being throughout the menstrual cycle [5]. This broader concept requires access to accurate information, affordable and clean menstrual products, adequate water and sanitation facilities, and a supportive environment free from stigma and discrimination [6,7]. When these conditions are not met, adolescent girls may experience shame, discrimination, reduced school attendance, and limited social participation [8].

Although menstruation has gained increasing attention globally as a public health and human rights issue, cultural taboos, misconceptions, and inadequate educational resources related to menstruation persist across many regions worldwide [9,10]. Studies from different cultural and social contexts have consistently shown that limited school-based education, reliance on informal information sources, and societal silence around menstruation contribute to poor menstrual hygiene knowledge and practices among adolescent girls [11,12,13,14].

Understanding adolescent girls’ knowledge of menstrual hygiene management and the factors influencing their practices is essential for developing culturally appropriate and context-specific interventions [14]. Evidence from Saudi Arabia indicates that menstruation remains a culturally sensitive topic, and many adolescent girls demonstrate gaps in knowledge and suboptimal menstrual hygiene practices, underscoring the need for targeted educational strategies [15]. However, available studies from Saudi Arabia are limited in number and geographic scope, leaving important regional variations insufficiently explored.

Accordingly, this study aimed to assess menstrual hygiene practices and identify their determinants among adolescent girls attending secondary schools in Bisha, Saudi Arabia. Guided by the Health Belief Model (HBM), the study examines sociodemographic characteristics, knowledge-related factors, and parental influences to address gaps in the existing literature and generate evidence to support school-based health education programs and community interventions that promote improved menstrual health and hygiene.

## 2. Materials and Methods

### 2.1. Study Design

The present study employed a quantitative, cross-sectional descriptive type to extensively explore the complex nature of menstrual hygiene behaviors and associated determinants among adolescent girls in secondary schools. In order to provide a strong and sufficient representation from different schools as well as grade levels, stratified random sampling was taken into account. The elaborate data generation process took place over four months, from September 2023 to December of the same year, after the authors received ethical clearance from the relevant institutional review board in order to present the ethical guidelines followed by researchers.

### 2.2. Theoretical Framework

The study was guided by the Health Belief Model (HBM), which provided a theoretical framework to examine factors influencing menstrual hygiene practices among adolescent girls. According to HBM, health behaviors are shaped by perceived susceptibility, perceived severity, perceived benefits, perceived barriers, cues to action, and self-efficacy. In this study, girls’ knowledge about menstruation reflects their perceived susceptibility and severity, while their attitudes and beliefs represent perceived benefits and barriers to maintaining proper hygiene. Cues to action were represented by education and guidance from mothers, teachers, and peers, whereas self-efficacy was reflected in participants’ confidence in their ability to manage menstruation independently and their age at menarche. Socio-demographic factors, including age, grade level, and mother’s education and occupation, served as modifying factors influencing these perceptions and behaviors. This theoretical framework guided the selection of study variables and provided a rationale for understanding variation in menstrual hygiene practices, explaining why some girls adopt recommended behaviors consistently while others do not.

### 2.3. Study Setting

The study was conducted in government secondary schools for girls in Bisha City, Asir region, Saudi Arabia, during the 2022–2023 academic year. Four schools were randomly selected from a total of seven eligible schools, and no schools were excluded due to administrative restrictions or other criteria. The selected schools represent typical urban and semi-urban educational settings in Bisha, allowing for a representative sample of adolescent girls in the district. Approximately 2100 girls were enrolled in grades one to three. Three hundred and twenty students were randomly sampled proportionally from class lists for inclusion in the study.

### 2.4. Study Participants

The sample comprised school-attending adolescent females aged from 15 to 19, studying in government secondary schools of Bisha City. Teenage girls were targeted, as adolescence is a crucial period for the formation of good menstrual hygiene practices. The recruitment of girls from multiple grades allowed us to examine practices at differing stages of adolescence. All participants were provided with information about the purpose of the study, and participation was voluntary and confidential. Exclusion criteria encompassed those who had not experienced menarche, refused to participate in the study, or were absent on the days of data collection.

### 2.5. Sample Size Determination and Sampling Procedure

The sample size was determined using a standard formula for finite population correction to ensure representativeness and precision:

n = [N × Z^2^ × p × (1 − p)]/[e^2^ × (N − 1) + Z^2^ × p × (1 − p)]

where N = 2100 (total population), Z1 − α/2 = 1.96 (for 95% confidence level), p = 0.431 (expected prevalence of good practice; Daniel et al., 2023) [16], and e = 0.05 (margin of error). The calculated sample size was 320 participants.

Stratified random sampling was used to ensure proportional representation across grades and schools. Four governmental secondary schools were randomly selected from seven eligible schools in Bisha. Within each selected school, the number of participants was allocated proportionally according to class size. Finally, students were randomly selected from class lists to obtain the final sample.

### 2.6. Data Collection Tool and Procedures

**Figure 1:**
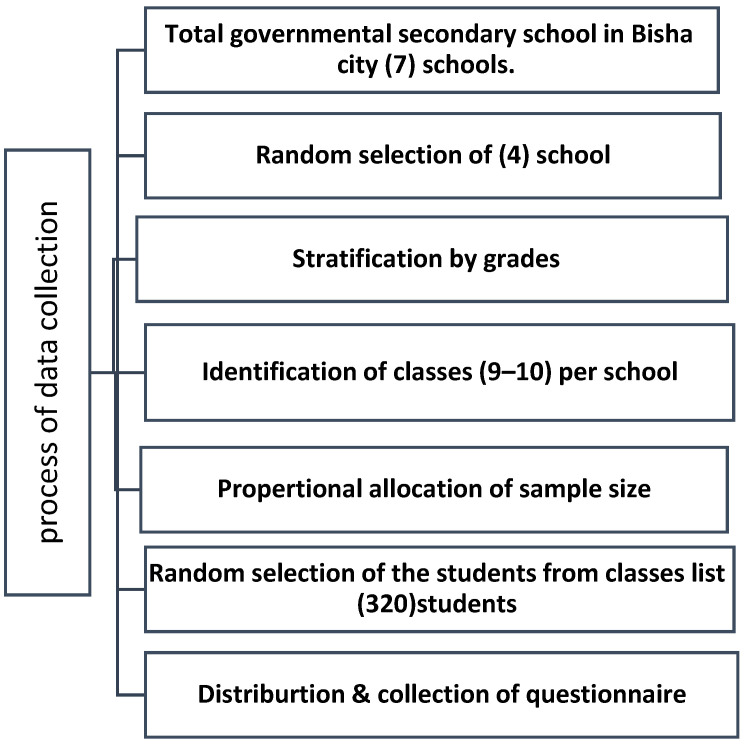
Flow diagram of the sampling and data collection process.

Data were collected using a structured, self-administered questionnaire adapted from previous studies [17,18,19]. The items were designed to capture knowledge and practices related to menstrual hygiene, reflecting constructs from the Health Belief Model (HBM), including perceived susceptibility, perceived severity, perceived benefits, perceived barriers, and self-efficacy regarding menstrual hygiene behaviors. The questionnaire comprised three sections: (1) sociodemographic characteristics (10 items), (2) knowledge regarding menstruation (11 items), and (3) menstrual hygiene practices (11 items). The survey was originally developed in English and then translated into Arabic using a professional bilingual translator. A process of back-translation was used to check the consistency of meaning. Items for knowledge and practice were scored as correct/Yes = 1 and incorrect/No = 0. Items for knowledge and practice were scored from 11, with scores of >6 considered “good” or ≤6 “poor” for category-wise outcomes [20]. Data were collected between September and December 2023 by an all-female research team. On scheduled data collection days, the research team visited each selected school during regular class hours after obtaining authorization from school administrators. Before administration, written consent was obtained from the parents or guardians, and assent was obtained from the participating students. During school visits, the study’s purpose, confidentiality, and voluntary participation were explained to students. Data collection was conducted in classrooms. Students completed the questionnaire independently. Completed questionnaires were checked immediately for missing items, and participants were asked to complete any unintentionally skipped questions. All questionnaires were collected on the same day, sealed in envelopes, and transported to the principal investigator for secure storage and data entry (Figure 1).

### 2.7. Validation Testing

Content validity was assessed by a panel of experts, including physicians and nursing researchers, who evaluated items for clarity, relevance, and cultural appropriateness. Based on their feedback, ambiguous items were rephrased, and culturally sensitive items were adapted to ensure suitability for the local context.

A pilot study was conducted with 32 adolescent girls who were selected from a school that was not included in the final study and was not included in the main sample. Participants provided feedback on item comprehension and response options, which resulted in minor wording adjustments and simplified instructions to ensure consistent understanding. These modifications did not alter the overall structure or scoring of the instrument. Internal consistency was assessed using Cronbach’s alpha, yielding satisfactory reliability for both the knowledge scale (α = 0.82) and the practice scale (α = 0.85), confirming that the questionnaire was suitable for assessing menstrual hygiene knowledge and practices in this population.

### 2.8. Data Analysis

Data were entered into EpiData version 3.1, checked for completeness, and exported to SPSS version 25 for analysis. Descriptive statistics (frequencies and percentages) summarized socio-demographic characteristics, knowledge, and practice levels. Bivariate analysis using chi-square tests identified associations between independent variables and menstrual hygiene practices. Variables with a *p*-value < 0.25 in bivariate analysis were included in a multivariable logistic regression model to determine independent predictors of good menstrual hygiene practices. The regression model was constructed using the enter method, where all selected variables were entered simultaneously. The results are presented as adjusted odds ratios (AORs) with 95% confidence intervals (CIs). Statistical significance was set at *p* < 0.05.

### 2.9. Ethical Considerations

Ethical approval was obtained from the Review Board of the Local Permanent Committee of Bioethics Research, University of Bisha, College of Medicine (Ref. No. UB-RELOC H-06-BH-087/(0405.23). It was conducted in accordance with the ethical principles outlined in the Declaration of Helsinki, including respect for voluntariness, confidentiality, and non-maleficence.

## 3. Results

### 3.1. Sample Descriptive Characteristics

Participants’ ages ranged from 15 to 19 years, with a mean age of 17.08 ± 1.0 years. 55% belonged to the 17–19-year age group. More than half of the participants, 53.8%, came from urban areas. In addition, 66.2% of the participants experienced menarche at 12 years of age or younger. A total of 27.5% and 29.4% of fathers and mothers had completed a university education. Moreover, a maximum of 30% of fathers were retired, while 43.4% of mothers were housewives. (Table 1).

### 3.2. Menstruation-Related Knowledge

Good knowledge of menstruation was observed in 52.5% of respondents. Overall, 37.8% indicated that menstruation is a normal physiological processes, and only 25.6% correctly stated that these are hormonal changes. Moreover, only 32.5% accurately identified that menstrual blood appears in the uterus, while 41.2% mistakenly believed that it appears in the vagina. Approximately two-thirds (64.4%) recognized the importance of maintaining personal hygiene during menstruation. Regarding absorbent materials, 34.4% identified sanitary pads as the most effective option. Only 20% knew that normal menstrual bleeding lasts 2–5 days, while 42.2% reported that it lasts more than 5 days. In addition, 62.2% and 59.4% agreed that menstrual blood is unclean and has an unpleasant odor, respectively. Moreover, 67.5% correctly recognized that menstruation is a temporary condition (Table 2).

### 3.3. Menstrual Hygiene Practices

Out of all participants, 53.8% exhibited good menstrual hygiene practices. During their most recent menstruation period, 37.5% of respondents reported using absorbent materials, while 73.8% used disposable sanitary pads. More than half (55.6%) bathed with water and soap during menstruation, though only 37.2% washed their hands before cleaning their genital area. Furthermore, 60.0% changed their pads more than three times per day, 68.8% disposed of used pads in dustbins, and 63.1% wrapped them in paper before disposal (Table 3).

### 3.4. Factors Associated with Menstrual Hygiene Practices

Several factors were significantly associated with good menstrual hygiene practices, including age, residence, age at menarche, parents’ occupation, and menstrual knowledge. Participants aged 17–19 years were 2.693 times more likely to report good menstrual practices than those aged 15–<17 (AOR: 2.693, 95% CI: 1.59–4.56). Urban residents were 2.62 times more likely to practice good menstrual hygiene than rural participants were (AOR: 2.62, 95% CI: 1.47–4.69). Girls with later menarche (after age 12) were less likely to practice good menstrual hygiene than those with earlier menarche (AOR = 0.489, 95% CI: 0.28–0.90).

Participants whose mothers worked in government jobs were 7.35 times more likely to report good menstrual hygiene than those whose mothers were housewives (AOR: 7.346, 95% CI: 2.29–23.54), and those with mothers in the private sector were 3.05 times more likely to report good menstrual hygiene (AOR: 3.051, 95% CI: 0.75–12.48). Furthermore, participants with employed fathers demonstrated significantly better hygiene practices than those with fathers in government or agricultural sectors. Importantly, participants with adequate knowledge were twice as likely to engage in healthy menstrual hygiene practices (AOR: 2.129, 95% CI: 1.236–3.666) (Table 4).

## 4. Discussion

This study assessed menstrual hygiene practices and their associated factors among adolescent secondary school girls in Bisha, Saudi Arabia. Overall, 53.8% of participants demonstrated good menstrual hygiene behavior, a proportion comparable to findings reported in other regions of Saudi Arabia [21,22]. Although slightly above average, this level still suggests persistent gaps in menstrual health education delivered through school curricula and community health programs [23,24].

Most girls (73.8%) reported using disposable sanitary pads during their last menstrual period, which reflects improving access to menstrual products and echoes trends in recent Saudi studies highlighting increased utilization of commercial sanitary materials among adolescents [22]. Despite these positive developments, financial constraints and culturally driven hesitations continue to hinder menstrual equity, indicating the need for more inclusive policies and community-level awareness initiatives.

However, several essential hygiene behaviors were suboptimal. Only 37.2% of girls reported washing their hands before cleaning their genital area, and more than half (55.6%) did not bath regularly during menstruation. These results represent persistent cultural taboos and misinformation surrounding menstrual purity. Similar patterns have been observed in studies from Egypt, Bangladesh, and Ethiopia, where menstrual misconceptions and cultural stigma hinder adequate menstrual hygiene practices [24,25,26].

Alarmingly, 44.4% of girls believed that menstruation occurs due to “past sins,” revealing a deeply rooted misconception. Such beliefs indicate a profound knowledge gap that can negatively shape girls’ psychological well-being, self-perception, and health behaviors. Misconceptions of this nature have been documented in other Middle Eastern contexts and are often perpetuated by silence around menstruation within families and schools [24]. These findings underscore the urgent need for culturally sensitive educational interventions targeting both adolescents and caregivers.

Additionally, low rates of handwashing before genital cleansing and the high proportion of girls (62.2%) who perceived menstrual blood as “unclean” indicate entrenched stigma that undermines healthy menstrual management. WHO and UNICEF emphasize that menstrual blood is a normal physiological fluid and that negative perceptions can lead to secrecy, shame, and avoidance of proper hygiene [27]. Addressing these misconceptions within school-based health programs could substantially improve health outcomes.

Sociodemographic characteristics played an important role in shaping menstrual hygiene behaviors. Older adolescents and urban residents exhibited significantly better menstrual hygiene practices, likely due to greater access to information, school health resources, and family guidance. This finding aligns with earlier work indicating that urban residence enhances opportunities for reproductive health education [27]. Maternal education, in particular, showed a strong association with good practices, reflecting the critical role mothers play in informing daughters about menstruation. Mothers with higher educational levels are more comfortable discussing reproductive issues openly, resulting in more informed daughters [28].

Cultural influences specific to Saudi Arabia—including religious modesty norms, the cultural sensitivity surrounding reproductive topics, and limited family dialogue—are also essential for understanding these patterns. Prior research has shown that girls in conservative contexts may experience restricted access to menstrual knowledge, fewer opportunities to discuss menstruation openly, and greater internalization of menstrual stigma [29]. These cultural elements, combined with school-level gaps, create barriers that shape the menstrual experiences of Saudi adolescents.

Knowledge emerged as a significant independent predictor of menstrual hygiene practices, consistent with evidence from national and international studies [15,30,31]. When girls have accurate menstrual knowledge, they are more likely to adopt safe and healthy practices. This reinforces the need for formal school-based reproductive health education programs supported by parental involvement.

The overall findings emphasize the importance of strengthening menstrual health education as part of adolescent health initiatives in Saudi Arabia. Comprehensive, culturally appropriate health education—delivered across schools, families, and community platforms—has the potential to correct misconceptions, reduce stigma, and empower adolescent girls to manage menstruation with dignity [30]. Additionally, ensuring the availability of affordable menstrual products and improving school sanitation infrastructure would further promote healthy and equitable menstrual experiences.

### 4.1. Implications for Adolescent Health

The wide-ranging nature of the inquiry has a number of important implications for healthy living for adolescents. First, the moderate levels of menstrual hygiene practices evident from the study illustrate the critical need for the implementation of focused and targeted measures to improve adolescent schoolgirls’ understanding and practical qualifications in menstrual health. Where possible, studies provide adolescent girls with an integrated and holistic understanding of menstrual health in school curricula as part of community programs and health services. In these ways, young girls will receive consistent and holistic education on this important aspect of health. It is critical to address the deeply entrenched cultural myths and misunderstandings about menstruation. Fostering a climate for learning and opportunity to study enables girls to gain more confidence and works to dissolve the misunderstanding associated with this natural process. Access to sanitary products and sanitation facilities is also critical. Most significantly, rural areas continue to indicate disproportionately inadequate MMIP, suggesting that special attention should be paid to these regions.

### 4.2. Limitations and Recommendations of the Study

This study has some methodological limitations. Self-reported data may introduce social desirability bias, and the cross-sectional design prevents causal inferences. Additionally, single-city sampling in Bisha limits the generalizability of findings to other regions with different socioeconomic or cultural contexts. To strengthen future research, studies could benefit from adopting established theoretical frameworks such as the Health Belief Model to guide variable selection and interpretation. Using validated measurement scales with demonstrated factorial validity would enhance construct validity, while expanding data collection to multiple sites would improve generalizability. Furthermore, incorporating qualitative methods could provide a deeper understanding of cultural beliefs and social norms that influence menstrual hygiene practices, enabling more culturally sensitive and effective interventions.

## 5. Conclusions

Although a substantial proportion of respondents reported good menstrual hygiene practices, notable gaps in knowledge and behaviors persist. Age, place of residence, maternal education, and menstrual knowledge were significant predictors of good practices, while later age at menarche was associated with a lower likelihood of practicing optimal hygiene. These findings highlight the importance of targeted interventions, including school-based menstrual health education, family and community engagement, and culturally tailored programs to address misconceptions and improve access to menstrual hygiene materials. Implementing such measures can support the health, well-being, and empowerment of adolescent girls in Saudi Arabia

## Figures and Tables

**Table 1:** Sociodemographic characteristics of the studied participants.

Variable	Category	Number (320)	%
Age in years	15–<17	148	46.2
	17–19	172	53.8
Residence	Rural	144	45.0
	Urban	176	55.0
Age of menarche	≤12 years old	212	66.2
	>12 years old	108	33.8
Mother’s education level	Illiterate	37	11.6
	Can read and write	57	17.8
	Primary	48	15.0
	Secondary	84	26.2
	University	94	29.4
Father’s education level	Illiterate	28	8.8
	Can read and write	63	19.7
	Primary	59	18.4
	Secondary	82	25.6
	University	88	27.5
Mother’s Occupation	Housewife	139	43.4
	Governmental employee	109	34.1
	Others	72	22.5
Father’s occupation	Retired	96	30.0
	Governmental employee	92	28.7
	Private	64	20.0
	Farmer	68	21.3

**Table 2:** Menstruation knowledge among studied participants.

Variable	Category	Number (320)	%
Menstruation	is a normal process for girls & women	121	37.8
	Do not know	199	62.2
Cause of menstruation	Do not know	60	18.8
	Hormone	82	25.6
	Past sins	142	44.4
	Disease	36	11.2
Origin of menstrual blood	Do not know	48	15.0
	Uterus	104	32.5
	Vagina	132	41.2
	Ovary	36	11.3
Important to take care of personal hygiene	Don’t know	30	9.4
	No	84	26.2
	Yes	206	64.4
Good absorbent during menstruation	Old cloths/towels	90	28.1
	Sanitary Pad	110	34.4
	Do not know	120	37.5
Length of normal menstrual bleeding	<2 days	42	13.1
	2–5 days	64	20.0
	>5 days	135	42.2
	Don’t know	79	24.7
Normal duration of the menstrual cycle	<20 days	60	18.7
	20–35 days	64	20.0
	>35 days	125	39.1
	Don’t know	71	22.2
Menstrual blood has a foul smell	No	121	37.8
	Yes	199	62.2
Menstrual blood is unhygienic	No	130	40.6
	Yes	190	59.4
Pain during menstruation does not mean that someone is sick	No	113	35.3
	Yes	207	64.7
Menstruation is not a lifelong process	No	104	32.5
	Yes	216	67.5
Overall knowledge about menstruation	Poor	152	47.5
	Good	168	52.5

**Table 3:** Menstrual health practice among studied participants.

Variables	Number (320)	%
Using absorbent materials during menstruation	120	37.5
Using commercial disposable sanitary pads	236	73.8
Taking a bath during menstruation with water and soap	178	55.6
Wash your hands before cleaning your genitals	119	37.2
Cleaning genitals after urinating/defecating	139	43.4
Cleaning genitals before changing the pad	166	51.9
Clean genital using water and soap	165	51.9
Changing pad more than 3 times per day	192	60.0
Cleaning reusable cloths with soap and water	181	56.6
Drying the reusable cloths in sunlight	160	50.0
Disposing of pads by wrapping them in paper	202	63.1
Disposing of used sanitary pads in dustbins	220	68.8

**Table 4:** Binary regression analysis for menstrual hygiene practice.

Variable	Category	Poor 148	Good 172	AOR CI: 95%	*p* Value
Age in years	15–<17	89 (60.1%)	59 (39.9%)	Ref	
	17–19	59 (34.3%)	113 (65.7%)	2.693 (1.59–4.56)	0.000 **
Residence	Rural	81 (56.3%)	63 (43.8%)	Ref	
	Urban	67 (38.1%)	109 (61.9%)	2.622(1.47–4.69)	0.001 **
Age of menarche	≤12 years old	88 (41.5%)	124 (58.5%)	Ref	
	>12 years old	60 (55.6%)	48 (44.4%)	0.489 (0.28–0.90)	0.022 *
Mother’s education level	Illiterate	28 (75.7%)	9 (24.3%)	Ref	0.069
	Can read and write	33 (57.9%)	24 (42.1%)	3.455 (0.79–15.02)	0.099
	Primary	20 (41.7%)	28 (58.3%)	8.033 (1.44–44.99)	0.018 *
	Secondary	31 (36.9%)	53 (63.1%)	5.642 (1.22–26.01)	0.026 *
	University	36 (38.3%)	58 (61.7%)	5.252 (1.33–20.71)	0.018 *
Father education level	Illiterate	16 (57.1%)	12 (42.9%)	Ref	0.136
	Can read and write	30 (47.6%)	33 (52.4%)	0.271 (0.06–1.23)	0.090
	Primary	30 (50.8%)	29 (49.2%)	0.153 (0.03–0.85)	0.032 *
	Secondary	32 (39.0%)	50 (61.0%)	0.331 (0.07–1.63)	0.175
	University	40 (45.5%)	48 (54.5%)	0.189 (0.05–0.78)	0.021 *
Mother’s occupation	Housewife	73 (52.5%)	66 (47.5%)	Ref	0.002 *
	Government employee	39 (35.8%)	70 (64.2%)	7.346 (2.29–23.54)	0.001 **
	Private employee	36 (50.0%)	36 (50.0%)	3.051 (0.75–12.48)	0.121
Father’s occupation	Retired	35 (36.5%)	61 (63.5%)	Ref	0.006 *
	Government employee	40 (43.5%)	52 (56.5%)	0.169 (0.05–0.61)	0.007 *
	Private employee	35 (54.7%)	29 (45.3%)	0.216 (0.05–0.98)	0.047 *
	Farmer	38 (55.9%)	30 (44.1%)	0.275 (0.12–0.61)	0.002 *
Knowledge	Poor	90 (59.2%)	62 (40.8%)	Ref	
	Good	58 (34.5%)	110 (65.5%)	2.129 (1.24–3.67)	0.006 *

* *p* < 0.05; ** *p* < 0.01; CI = confidence interval; AOR = adjusted odds ratio.

## Data Availability

Data are available upon request due to privacy restrictions.

## References

[1] Wegner M., Amatriain-Fernández S., Kaulitzky A., Murillo-Rodriguez E., Machado S., Budde H. (2020). Systematic Review of Meta-Analyses: Exercise Effects on Depression in Children and Adolescents. Front. Psychiatry.

[2] Sommer M., Torondel B., Hennegan J., Phillips-Howard P.A., Mahon T., Motivans A., Zulaika G., Gruer C., Haver J., Caruso B.A. (2021). Monitoring Menstrual Health and Hygiene Group. How addressing menstrual health and hygiene may enable progress across the Sustainable Development Goals. Glob. Health Action.

[3] UN Women (2018). Turning Promises into Action: Gender Equality in the 2030 Agenda for Sustainable Development.

[4] Patel K., Panda N., Sahoo K.C., Saxena S., Chouhan N.S., Singh P., Ghosh U., Panda B. (2022). A systematic review of menstrual hygiene management (MHM) during humanitarian crises and/or emergencies in low- and middle-income countries. Front. Public Health.

[5] Hennegan J., Winkler I.T., Bobel C., Keiser D., Hampton J., Larsson G., Chandra-Mouli V., Plesons M., Mahon T. (2021). Menstrual health: A definition for policy, practice, and research. Sex. Reprod. Health Matters.

[6] Deshpande T.N., Patil S.S., Gharai S.B., Patil S.R., Durgawale P.M. (2018). Menstrual hygiene among adolescent girls—A study from urban slum area. J. Fam. Med. Prim. Care.

[7] Marques P., Madeira T., Gama A. (2022). Menstrual cycle among adolescents: Girls’ awareness and influence of age at menarche and overweight. Rev. Paul. Pediatr..

[8] van Eijk A.M., Zulaika G., Lenchner M., Mason L., Sivakami M., Nyothach E., Unger H., Laserson K., Phillips-Howard P.A. (2019). Menstrual cup use, leakage, acceptability, safety, and availability: A systematic review and meta-analysis. Lancet Public Health.

[9] Hennegan J., Shannon A.K., Rubli J., Schwab K.J., Melendez-Torres G.J. (2019). Women’s and girls’ experiences of menstruation in low- and middle-income countries: A systematic review and qualitative metasynthesis. PLoS Med..

[10] Mathiyalagen P., Peramasamy B., Vasudevan K., Basu M., Cherian J., Sundar B.A. (2017). descriptive cross-sectional study on menstrual hygiene and perceived reproductive morbidity among adolescent girls in a union territory, India. J. Fam. Med. Prim. Care.

[11] van Lonkhuijzen R.M., Garcia F.K., Wagemakers A. (2022). The Stigma Surrounding Menstruation: Attitudes and Practices Regarding Menstruation and Sexual Activity During Menstruation. Women’s Reprod. Health.

[12] Barrington D.J., Robinson H.J., Wilson E., Hennegan J. (2021). Experiences of menstruation in high income countries: A systematic review, qualitative evidence synthesis and comparison to low- and middle-income countries. PLoS ONE.

[13] Joshi K., Mendhe D. (2025). Navigating Menstrual Health and Hygiene: Challenges and Solutions for Adolescent Girls. J. Pharm. Bioallied Sci..

[14] Sommer M., Caruso B.A., Torondel B., Warren E.C., Yamakoshi B., Haver J., Long J., Mahon T., Nalinponguit E., Okwaro N. (2021). Menstrual hygiene management in schools: Midway progress update on the “MHM in Ten” 2014–2024 global agenda. Health Res. Policy Syst..

[15] Al Mutairi H., Jahan S. (2021). Knowledge and practice of self-hygiene during menstruation among female adolescent students in Buraidah city. J. Fam. Med. Prim. Care.

[16] Daniel N., Kejela G., Fantahun F., Desalegn M., Guteta F. (2023). Menstrual hygiene management practice and its associated factors among in-school adolescent girls in Western Ethiopia. Contracept. Reprod. Med..

[17] Abita Z., Ali R., Admassu B. (2021). Menstrual hygiene management practice and associated factors among secondary school girls in finot selam town, northwest Ethiopia, 2019. Int. J. Sex. Reprod. Health Care.

[18] Mohammed Gena H. (2020). Menstrual hygiene management practices and associated factors among secondary school girls in East Hararghe Zone, Eastern Ethiopia. Adv. Public Health.

[19] Ha M.A.T., Alam M.Z. (2022). Menstrual hygiene management practice among adolescent girls: An urban-rural comparative study in Rajshahi division, Bangladesh. BMC Women’s Health.

[20] Ngilangwa D.P., Ngalesoni F., Temu F., Meremo A.J., Cooper D., Lembani M. (2025). Menstrual Hygiene Management Practices and Associated Factors Among Adolescent Schoolgirls in Rural Tanzania: A School-Based Cross-Sectional Study. Women’s Reprod. Health.

[21] Alharbi K.K., Alkharan A.A., Abukhamseen D.A., Altassan M.A., Alzahrani W., Fayed A. (2018). Knowledge, readiness, and myths about menstruation among students at the Princess Noura University. J. Fam. Med. Prim. Care.

[22] Alenizy H., Aleyeidi N., Almutairi R., Khosyfan L., Bedaiwi R., Alowaidah L., Alrushud H., Alfadda K., Alshamekh L.A., Al Anazi N. (2024). Assessment of the Readiness, Beliefs, and Practices Regarding Menstruation Among Women in Saudi Arabia. Int. J. Women’s Health.

[23] Ayele A., G/Mariam M., Beyene H., Tolcha A., Tediso D., Shalamo T., Belayneh T. (2025). Menstrual hygiene practice and associated factors among adolescent primary school females in Dale Woreda, Sidama, Ethiopia: A cross sectional study. Front. Reprod. Health.

[24] El-Gilany A.H., Badawi K., El-Fedawy S. (2005). Menstrual hygiene among adolescent schoolgirls in Mansoura, Egypt. Reprod. Health Matters.

[25] Alam M.U., Luby S.P., Halder A.K., Islam K., Opel A., Shoab A.K., Ghosh P.K., Rahman M., Mahon T., Unicomb L. (2017). Menstrual hygiene management among Bangladeshi adolescent schoolgirls and risk factors affecting school absence: Results from a cross-sectional survey. BMJ Open.

[26] Melaku A., Addis T., Mengistie B., Kanno G.G., Adane M., Kelly-Quinn M., Ketema S., Hailu T., Bedada D., Ambelu A. (2023). Menstrual hygiene management practices and determinants among schoolgirls in Addis Ababa, Ethiopia: The urgency of tackling bottlenecks—Water and sanitation services. Heliyon.

[27] Belayneh Z., Mekuriaw B. (2019). Knowledge and menstrual hygiene practice among adolescent school girls in southern Ethiopia: A cross-sectional study. BMC Public Health.

[28] Korir E., Okwara F.N., Okumbe G. (2018). Menstrual hygiene management practices among primary school girls from a pastoralist community in Kenya: A cross sectional survey. Pan Afr. Med. J..

[29] Mohammed S., Larsen-Reindorf R.E. (2020). Menstrual knowledge, sociocultural restrictions, and barriers to menstrual hygiene management in Ghana: Evidence from a multi-method survey among adolescent schoolgirls and schoolboys. PLoS ONE.

[30] Alsalami O.A., Kamel S., Almatrafi R.S., Alalwani B.M., Alanazi A.I., Algarni A.D., Almatrafi N.S., Alsalami F.A., Algarni M.D., Almatrafi M.S. (2025). Enhancing Self-Hygiene Awareness and Practices During Menstruation Among Female Adolescent Students in Saudi Arabia: A Comprehensive Educational Program Initiative. Cureus.

[31] Wasan Y., Baxter J.B., Rizvi A., Shaheen F., Junejo Q., Abro M.A., Hussain A., Ahmed I., Soofi S.B., Bhutta Z.A. (2022). Practices and predictors of menstrual hygiene management material use among adolescent and young women in rural Pakistan: A cross-sectional assessment. J. Glob. Health.

